# Bioactivity, Efficacy, and Safety of a Wound Healing Ointment With Medicinal Plant Bioactives: In Vitro and In Vivo Preclinical Evaluations

**DOI:** 10.1155/tswj/9466270

**Published:** 2025-02-26

**Authors:** Juliana Ferreira Floriano, Daniel Rodrigues, Rie Ohara, Nara Lígia Martins Almeida, Vanessa Soares Lara, Patricia Sartorelli, Carlos Frederico de Oliveira Graeff, Simone dos Santos Grecco, Alejandra Hortencia Miranda González, Paulo Henrique Perlatti D'Alpino

**Affiliations:** ^1^Imperial College London, National Heart and Lung Institute, London, UK; ^2^Botucatu Medical School, São Paulo State University (UNESP), Botucatu, São Paulo, Brazil; ^3^Bioengineering and Biomaterials Group, School of Pharmaceutical Sciences, São Paulo State University (UNESP), Araraquara, São Paulo, Brazil; ^4^Biotechnology and Innovation in Health Program, Universidade Anhanguera de São Paulo (UNIAN), São Paulo, Brazil; ^5^Department of Health Management, Fundação Nacional de Gestão de Saúde, São Paulo, Brazil; ^6^Undergraduate Program, Medical School, Universidade Nove de Julho, Bauru, São Paulo, Brazil; ^7^Department of Surgery, Stomatology, Pathology and Radiology, Bauru School of Dentistry, University of São Paulo (USP), Bauru, São Paulo, Brazil; ^8^Department of Chemistry, Institute of Environmental, Chemical and Pharmaceutical Sciences, Federal University of São Paulo, Diadema, São Paulo, Brazil; ^9^Department of Physics, School of Sciences, POSMAT-Post-Graduate Program in Materials Science and Technology, São Paulo State University (UNESP), Bauru, São Paulo, Brazil; ^10^Department of Research and Development (R&D) and Innovation Center, Triplet Biotechnology Solutions, Inc., Bauru, São Paulo, Brazil; ^11^Post-Graduate Program in Dentistry, Uniderp Anhanguera University, Campo Grande, Mato Grosso do Sul, Brazil; ^12^Post-Graduate Program in Integrated Dental Sciences, University of Cuiabá (UNIC), Cuiabá, Mato Grosso do Sul, Brazil; ^13^Department of Physics, School of Sciences, São Paulo State University (UNESP), Bauru, São Paulo, Brazil

**Keywords:** biocompatibility, chronic wounds, natural compounds, preclinical tests, wound care

## Abstract

Chronic wounds have a significant impact on patients' quality of life, necessitating the management of pain, infection, bleeding, and emotional challenges. Debridement, which involves the removal of nonviable tissue, is crucial for promoting wound healing. In addition to surgical methods, cost-effective alternatives such as local solutions and ointments with biological properties have been explored. The use of natural compounds with anti-inflammatory, antibacterial, and collagen-synthesizing abilities holds promise for wound healing. This in vitro and in vivo preclinical study aimed to assess the safety and effectiveness of a wound healing ointment containing bioactive ingredients derived from medicinal plants (extracts, essential oils, and vegetable oils). The chemical composition of the ointment was characterized using Fourier transform infrared (FTIR) spectroscopy to gain insights into its synergistic action. Preclinical tests were conducted following standardized protocols. FTIR analysis revealed similarities between the product's spectrum and that of bioactive compounds. The in vitro tests demonstrated that all formulations of the ointment induced no cell death, DNA damage, or acute toxicity in cell cultures (*p* < 0.05). No lethal dose was observed, indicating the safety of the ointment at all concentrations. The ointment also stimulated a notably more organized, significantly higher collagen production compared to control groups (*p* < 0.05). In vivo preclinical analyses also demonstrated no adverse responses being effective in the healing process compared to the control group (silver sulfadiazine) in terms of wound contraction and ulcer re-epithelization (*p* < 0.05). Significantly higher means of wound contraction were observed in the groups treated with the bioactive-containing ointment when compared to both the positive control group (sulfadiazine) and the control untreated groups (*p* < 0.05). The regenerative ointment exhibited excellent biocompatibility and bioactivity in in vitro and in vivo studies, contributing to the development of innovative and sustainable wound management therapies.

## 1. Introduction

Chronic wounds can have a significant impact on quality of life, comparable to that of kidney and heart disease [1]. These persistent wounds, caused by various conditions such as diabetes, fail to heal within a reasonable timeframe, leading to pain and disability [2]. Currently, it is observed that the mortality rate of patients with chronic wounds is similar to that of patients with cancer [3]. Chronic wounds, such as ulcers in the lower limbs, constitute a serious public health problem, affecting a large portion of the population, mainly adults and the elderly [4]. Local treatment aims to alleviate pain and itching, prevent wound infection and bleeding, and address the physical and emotional challenges associated with chronic wounds. These challenges include managing excessive exudate, which can result in unpleasant odors [5]. Chronic wounds interfere with patients' quality of life, as well as with morbidity and mortality rates, as they produce chronic changes in skin integrity, often causing incapacitation and/or amputation of lower limbs in these individuals [6].

Chronic skin wounds can be classified, according to their etiology, into venous, arterial, mixed, and neuropathic, among others, and have their own diagnostic characteristics [7]. Health professionals play a crucial role in diagnosing and distinguishing various wound types to recommend appropriate treatments. Clinical evaluation is vital for identifying intrinsic factors that impact wound healing. Moreover, numerous clinical conditions, including advanced age, diabetes mellitus, chronic renal failure, obesity, malnutrition, and connective tissue disorders, increase the risk of infection [8]. Each wound should be assessed individually, taking into account factors such as the time and cause of injury as well as the wound characteristics and patient factors that can influence the functional and esthetic outcomes [9].

Debridement of the wound bed is a crucial step in facilitating enhanced wound healing [10]. This technique involves the partial or complete removal of nonviable tissue from the wound, aiding in the preparation of the wound bed and promoting healing [11]. By eliminating devitalized or necrotic tissues, debridement supports the regeneration of healthy tissue and improves the overall healing process [10]. While surgical methods are commonly used for debridement, there are cost-effective alternatives that involve prescribing associated methods, such as the application of local solutions and ointments with autolytic, enzymatic, and/or biological action, along with mechanical removal techniques like simple curettage. These approaches offer effective means to facilitate debridement without the need for extensive surgical procedures, providing more accessible options for wound treatment [11].

In this context, there is a growing interest in exploring innovative approaches to debridement by investigating the potential of natural compounds [12]. These compounds possess valuable properties such as anti-inflammatory, antioxidant, antibacterial, and collagen-synthesizing abilities, making them promising candidates for effective wound healing agents. This pursuit of natural compounds reflects the ongoing quest for alternative and sustainable solutions in the field of wound care, driving research and development toward novel therapeutic interventions [13]. Numerous studies have been conducted to evaluate the efficacy of these compounds, opening new avenues for the development of novel therapies in wound management [5, 14–17]. The therapeutic effects of natural products can be attributed to the presence of bioactive phytochemical components such as alkaloids, saponins, and phenolic compounds, including flavonoids and tannins, and also obtained from essential oils [18]. Therefore, the aim of this in vitro study was to systematically assess the safety and efficacy of a wound healing ointment containing bioactives derived from medicinal plants, including extracts and essential and vegetable oils, along with other ingredients. Preclinical tests, including cytotoxicity, genotoxicity, and acute toxicity assessments, were conducted based on ISO 10993-3 and ISO 10993-5 [19, 20]. An in vivo preclinical test was also performed to analyze the influence of this wound healing ointment on the healing of excisional skin wounds using animal model. Additionally, by Fourier transform infrared (FTIR) spectroscopy, a physicochemical characterization of the bioactives present in the formulation and ingredients was performed to gain a comprehensive understanding of their composition and their synergistic action in the final formulation. Both in vitro and in vivo preclinical studies confirmed the efficacy and safety profile of the tested ointment formulations, which caused no excessive cell death, membrane damage, DNA damage, or acute toxicity and caused no adverse responses, being effective in the healing process, similar to the gold standard (silver sulfadiazine). However, limitations exist as in vitro conditions and can differ from in vivo and clinical settings. Future research should focus on clinical studies to validate their efficacy and assess long-term effects, interactions, and clinical impact on wound healing.

## 2. Materials and Methods

### 2.1. Product Description

Pharmacure^+^ Classic Reestruturante (TAG Distribuição e Comercialização de Importados em Geral Ltda, Belo Horizonte, Brazil) is a skin wound healing ointment that contains bioactives from medicinal plants, including extracts and essential and vegetable oils, in association with other bioactive ingredients. In Table 1, the bioactives present in the product formulation are listed.

In order to assess the safety of the product, three different variations were tested according to the concentrations of bioactives, namely, PHP (standard concentration); PHP+ (three times higher bioactives than the standard); and PHP− (three times lower bioactives than the standard). The products were kept at room temperature and away from light until the moment of the study.

### 2.2. FTIR Spectroscopy Analysis of the Chemical Bonds

The chemical bonds present in the formulations (PHP, PHP+, and PHP-) as well as in the ingredients were analyzed using spectroscopic technique. For that, FTIR analysis was performed using an IRPrestige-21 FTIR Spectrometer (Shimadzu Corporation, Kyoto, Japan). Infrared spectra were obtained by placing each sample directly against the diamond ATR crystal. Data were collected in transmission mode using 64-scan accumulation at a resolution of 4 cm^−1^. All data were processed using the OriginPro 8 software (OriginLab Corporation Northampton, MA, USA). The preprocessing data analysis was applied to the infrared spectral range from 4000 to 400 cm^−1^. Then the optimization of a range of preprocessing techniques was undertaken, namely, baseline correction, normalization (standard normal variate, vector, and min–max), and de-noising with smoothing of 1^st^ and 2^nd^ derivatives. The chemical bonds were determined by comparing the recorded spectra with the standard spectral library.

### 2.3. Cell Culture

The NIH/3T3 strain (ATCC CRL-1658™) was used, corresponding to mouse embryonic fibroblast cells purchased from the American Type Culture Collection (ATCC, Rockville, Maryland, USA). The NIH/3T3 strain was cultivated in DMEM culture medium (Dulbecco's Modified Eagle Medium, Gibco®, Thermo Fisher Scientific, São Paulo, Brazil) supplemented by 10% fetal bovine serum (FBS) (HyClone®, Karnataka, India) and 1% penicillin/streptomycin (Sigma-Aldrich®, São Paulo, Brazil). Cells were observed under an inverted microscope (Olympus® CKX41, Tokyo, Japan), and the culture flask was kept in an oven at 95% relative humidity, 5% CO_2_, and 37°C (Ultra Safe® HF 212 UV model, Heal Force, China) [21, 22].

### 2.4. Subculture and Standardization

Cell growth was monitored every 24 h using an inverted phase microscope (Olympus® CKX41, Tokyo, Japan). Characteristics such as cell confluence, cell morphology, cell proliferation, cell–cell interactions, cell viability, and cell migration were investigated. The culture medium was changed every 48 h to remove dead cells and nourish live cells. Fibroblasts are cells that grow in an adherence monolayer, and after approximately 80% confluence in the culture flask, subculturing was carried out. The culture medium was discarded, and the cells were washed with 5 mL of phosphate-buffered saline (PBS) three times. Then, 3 mL of 0.25% trypsin solution (HyClone®, Karnataka, India) was added to the culture flask to dissociate the cells at the bottom of the flask. Afterward, 3 mL of supplemented medium was added for trypsin inactivation. The solution was centrifuged at 1200 rpm for 5 min at 21°C. The supernatant was discarded, and the pellet was resuspended in 1 mL of medium. The fibroblasts were divided into two or more culture flasks containing 10 mL of supplemented medium until the medium was changed to create a new subculture. For standardization, after this process and the resuspension of the pellet, a 1:10 dilution was made (pellet: medium) and an aliquot of the 10 μL dilution was placed in a Neubauer chamber. The counting process was performed in multiple areas of the Neubauer chamber and the average calculated in order to improve accuracy for cell quantification. A common optical microscope (DM4 M, Leica Microsystems, Ltda®, São Paulo, SP, Brazil) was used for the acquisition of 5 x 10^3^ cells per well, for 96-well plates, which were kept in a humid incubator for 48 h in order to guarantee the adhesion of the cells by the entire well (100% confluence).

### 2.5. Cytotoxicity Assessment

An aliquot (500 mg) of the wound healing ointment, considering its three different formulations (PHP, PHP+, and PHP-), was added to centrifuge tubes containing 3 mL of supplemented medium (DMEM + 10% FBS + 1% penicillin/streptomycin). Specimens were incubated in an oven at 95% relative humidity, 5% CO_2_, and 37°C (Ultra Safe® Model HF 212 UV) for 24 h, producing, at the end of the incubation, the corresponding conditioned medium.

The conditioned medium was then diluted in a 1:1 ratio (conditioned media:supplemented media only) [23], and subsequently 200 μL of the diluted conditioned medium solutions was used in indirect contact with the cells in each of the analyses performed, constituting the experimental groups PHP, PHP+, and PHP−. The 1:1 dilution ratio is typically chosen because it provides a balanced environment for cells, ensuring that the components of the conditioned medium are present in optimal concentrations to support accurate and reliable results in cell-based assays such as XTT. The pH of each conditioned medium was measured on a universal pH strip (pH Strip, pH-fix 0–14, FortLab Express, Rio de Janeiro, Brazil). As a negative control group, supplemented DMEM medium was used (the medium group), and as a positive control, sterile distilled water was used (Water group). After 24, 48, and 72 h, the plates were washed 1x with PBS, and the XTT assay was performed (*n* = 5) according to a previous study [24], with modifications [25].

### 2.6. Cell Viability Assay

The study design involved the evaluation of fibroblast NIH/3T3 proliferation in indirect contact with the product by means of an XTT assay. The XTT assay is a colorimetric test used to measure cell viability, proliferation, and cytotoxicity. It assesses the metabolic activity of cells by evaluating their ability to reduce the salt, 2,3-bis(2-methoxy-4-nitro-5-sulfophenyl)-2H-tetrazolium-5-carboxanilide (Sigma-Aldrich Inc., St. Louis, MO, USA), to a soluble formazan product. The amount of formazan produced is proportional to the number of viable cells, as it reflects cellular metabolic activity, specifically the activity of mitochondrial enzymes. The color intensity is measured using a spectrophotometer, with higher absorbance indicating greater cell viability or metabolic activity. The XTT salt (Sigma Aldrich® Inc., St. Louis, MO, USA) was dissolved in saline solution at a concentration of 1 mg/mL. Menadione (2-Methyl-1,4-naphthoquinone, Sigma-Aldrich Inc., St. Louis, MO, USA) was dissolved in acetone at a concentration of 1 mmol/L. The formed reagent, XTT/menadione, was prepared before each assay. After the incubation time (24, 48, and 72 h) and the washing of the plates, 200 μL of XTT/menadione was added in triplicate, and the plates were kept in an oven at 95% relative humidity, 5% CO_2_, and 37°C (Ultra Model Safe HF 212 UV) for 3 h in the dark. Afterward, the reading was performed in a spectrophotometer (Monochromator® based on Biotek Synergy MX, Winooski, VT, USA), and the absorbance at a wavelength of 490 nm was evaluated. The percentage reduction in metabolic activity of viable cells was calculated as percentages of optical density (OD) in the wells containing the medium group (representing 100% metabolic activity). Three independent experiments were performed for each induction period. According to the international standard ISO 10993-5 [19], which outlines the standards for in vitro cytotoxicity testing of medical devices, a reduction in cell viability greater than 70% is considered positive for cytotoxicity.

### 2.7. Evaluation of % Cell Viability in Cultures Exposed to Wound Healing Ointment (XTT Analysis)

The cell proliferation assay was performed according to the ISO 10993-5 standard [19]. Briefly, 2 × 3 mm of each sterile sample was placed into 24-well plates. According to the protocol procedure, 1 mL of fibroblast medium was added (hereafter referred to as “extraction medium”) to each well and incubated for 24 h at 37°C. Then, under standard cell culture conditions, 10^3^ cells per well were seeded in 100 μL of extraction medium. The negative control group was seeded in 100 μL of fibroblast medium. The XTT assay (Cayman Chemical, Ann Arbor, MI, USA) allowed observation of the fibroblasts' starting condition (T0) and proliferation activity at 24 h (T1), 72 h (T2), and 7 days (T3) follow-ups at an absorbance wavelength of 490 nm. XTT tests were performed with three technical replicates.

### 2.8. Genotoxicity Assay—Comet Assay

To evaluate the genotoxicity of the samples, the comet assay was used, which allows the detection of damage in the DNA of a cell. The cultured cells that remained in contact with the samples (PHP, PHP+, and PHP−) for a period of 72 h were collected and submitted to the test, which was performed under alkaline conditions according to a previously described protocol [26]. For that, a volume of 5 μL of the suspension with cells was mixed with 100 μL of low melting point agarose (0.5%, Invitrogen Ltda, USA) dissolved in PBS (Invitrogen Ltda, USA) and spread onto microscope slides precoated with normal melting point agarose (1.5%, Invitrogen Ltda, USA). The slides were immersed for 24 h in a freshly prepared lysis solution (pH 10) consisting of 2.5 M NaCl, 100 mM ethylenediaminetetraacetic acid (EDTA, Sigma-Aldrich Co, São Paulo, Brazil), 10% dimethylsulfoxide (Merck Chemicals, São Paulo, Brazil), 1% Triton X-100 (Sigma-Aldrich Co.), and 10 mM Tris (Sigma-Aldrich Co, São Paulo, Brazil). After this period, the material was washed in PBS buffer and placed in a horizontal electrophoresis tank (28 × 17.5 × 6.5 cm, Técnica Permatron Ltda, Santa Catarina, Brazil) containing alkaline buffer (0.3 M NaOH, 1 mM Na_2_EDTA, pH > 13) at 4°C for 20 min.

Using the same buffer, electrophoresis was run for 20 min at an electric field strength of 1 V/cm (25 V and 300 mA). Subsequently, the slides were washed in a neutralization buffer (0.4 M Tris-HCl, pH 7.5) for 15 min, fixed for 5 min in absolute alcohol, air-dried, and stored at room temperature. The slides were stained with 50 μL of SYBR Green I (SGI) as a fluorescence probe, and examined with a magnification of 400x in a fluorescence microscope coupled to an image analysis system (Comet II; Perspective Instruments, Suffolk, UK). The visual scoring of the comet assay was evaluated by a single person to minimize scoring variation. All slides were coded, and 50 nucleotides were randomly analyzed.

The tail intensity (amount of DNA in the comet's tail) and the tail moment (product of tail length and the percentage of DNA in the tail) were used to measure the extent of DNA damage [27]. Comet images with a “cloudy” appearance or with a very small head and balloon-like tail were excluded from the analysis [26]. In addition, the early assessment of cytotoxicity was evaluated using this technique, which allows early identification of programmed cell death (apoptosis) by detecting DNA fragmentation in the cell nucleus and the genotoxic response induced by the products (PHP, PHP+, and PHP−). In this manner, this analysis demonstrates their safety in relation to possible gene mutations induced by the treatment [28].

### 2.9. In Vitro Cell Migration/Proliferation Assay

For the migration test, an in vitro scratch assay was used. For that, fibroblasts from NIH/3T3 were plated in a 96-well plate, and after the culture reached ∼100% confluence. Then, a linear scratch, mimicking an artificial wound, was created in the cellular monolayer using a sterile 200 μL plastic tip (usually after 18–24 h). The tip was kept perpendicular to the bottom of the well. Another scratch line was performed perpendicular to the first line to create a cross in each well. The tip was maintained in contact with the bottom of the well to remove the cell layer when gently making a scratch. The wells were then washed with sterile PBS to remove dead cells, and, subsequently, culture medium was supplemented with the products tested according to the experimental groups (PHP, PHP+, and PHP−). The well images with 4x magnification were acquired at 0, 24, 48, and 72 h, counting from the moment of the scratch, which corresponds to 0 h [16]. Images of scraped areas were acquired using an inverted microscope coupled with a camera (model INV-100, Leica Microsystems, Ltda®, São Paulo, Brazil).

Wound area recovery was measured using an image processing software (ImageJ/Fiji®, NIH, USA, https://imagej.nih.gov). In each trial, at least three replicates were performed. A negative control was used, which was composed of only cells in culture under untreated, normal conditions [29]. In addition, two positive controls (positive control 1: 1 μM colchicine, diluted with water; positive control 2: 1 μM colchicine, diluted with supplemented culture medium, DMEM) were used.

### 2.10. In Vitro Acute Toxicity Test—Up and Down

The IC_50_ test, which determines the cytotoxicity of a chemical in terms of the chemical's ability to inhibit the growth of half of a population of cells, was analyzed [30]. To calculate the IC_50_ of the experimental groups for the cytotoxicity evaluation, cells were exposed to different concentrations of the tested ointment concentrations (PHP, PHP+, and PHP−). Cytotoxicity analysis was performed according to the protocol previously described. Three replicates (*n* = 3) were performed in this test. The IC_50_ was calculated from curves constructed by plotting cell survival (%) *vs.* bioactive concentrations (μM). The reading values were converted to the percentage of the control (percentage cell survival). Cytotoxic effects were expressed as IC_50_.

### 2.11. Wound Healing Evaluation in Rat Model of Skin Wound

The animals used in the experiment were treated according to the instructions approved by the institutional review committee (Certificate 1421/2022–CEUA, Universidade Estadual Paulista–UNESP Botucatu). Animals were never exposed to inappropriate pain. For the study, 20 male rats weighing from 250 to 300 g were selected, from the Sprague Dawley® Specific Pathogen Free (SPF) line, aged 90 days (adults), obtained from the Bioterism Center of the Multidisciplinary Center for Biological Research at the State University of Campinas (CEMIB-UNICAMP), São Paulo, Brazil. The animals underwent an acclimatization period of approximately 60 days before the start of the experiments, in which they all received water and food (Nuvilab, Nuvital® Nutrientes S/A, Paraná, Brazil) ad libitum and were maintained (2 animals/box) under controlled conditions of temperature (22 ± 3°C), humidity (50 ± 10%), and light/dark cycle (12 h).

The animals were randomly distributed into four experimental groups (*n* = 5): three different formulations (Group 1: PHP, Group 2: PHP+, and Group 3: PHP-) and a group (Group 4) receiving 1% silver sulfadiazine cream (Dermazine®, brand Cristália, expiration date: 03/2025 and batch: 22630396, São Paulo, SP, Brazil). Group 4 was designated the gold standard or positive control, which refers to a commercial treatment or product commonly applied in clinical practice that presents satisfactory results for the treatment of the pathology for which it is intended. Silver sulfadiazine 1% is used to create a barrier between a wound and the environment as well as provide antibacterial properties which allow for re-epithelization and healing [31].

In all groups, two lesions measuring 2 cm in diameter and 5 cm apart were made on the back of the animals; the first cranial lesion, 7 cm away from their neck, received treatment with different variations of the Pharmacure + Classic Reestruturante product or the standard gold (treated groups). The second caudal lesion received no treatment other than its own blood clot (negative control). In this manner, in the same animal, both treated groups and a negative control (without treatment) were obtained in order to compare the outcomes of the ointment's action on the healing process and inflammatory response.

To perform the skin ulcer, the animals were previously anesthetized, according to the protocol approved by the ethics committee. The dorsal region was trichotomized and subsequently cleaned with povidone-iodine and saline solution after cleaning the center of the area. After depilation, the skin of each rat was demarcated by rotating the cutting edge of a 2 cm-diameter metal punch, and then the circular skin segment was resected, following the punch demarcation, deepening the incision until it exposed the dorsal muscular fascia. After performing the lesion, approximately 1 mL of each product was applied to the lesion corresponding to the treatment. To maintain consistency across animals, reduce experimental variability, and ensure reproducibility of the results, a sterile applicator was used to achieve uniform spreading and volume accuracy of the ointment when applying it to the wounds, ensuring even coverage. In the control untreated groups, wound healing relied on blood clot formation and natural physiological processes. These were carefully controlled by standardizing the wound creation technique, minimizing external interference, and providing a sterile environment to prevent infection. The ointments were applied every 12 h for 60 days. For post-injury analgesia, morphine was administered subcutaneously at a dose of 2.0 mg/kg every 4–6 h for 3 consecutive days, as approved by the ethics committee.

ImageJ software version 1.50i (https://imagej.net/ij/index.html) was used to analyze the wound area of each animal, as reported by Masson-Meyers et al. [32], in order to calculate the area of each wound over the experimental period. In the event of formation of erythema and bedsores, edema, bleeding, exudate, and other complications, these parameters were registered.

### 2.12. Wound Contraction Percentage (WCP)

WCP is calculated by comparing the area of the wound before and after treatment (*n* = 5). This helps assess how much the wound has closed or shrunk over time [33].

(1) $$\begin{array}{c} \text{WCP}=\left(1-\frac{A_{i}}{A_{f}}\right)\times 100, \end{array}$$

where *A*_*i*_ represents the initial area before healing and *A*_*f*_ is the final area after healing. The result of this calculation represents the percentage at which the wound contracted from its original size.

### 2.13. Ulcer Healing Rate (UHR)

In the same way, UHR is calculated by comparing the area of the wound before and after treatment (*n* = 5), according to the following equation:

(2) $$\begin{array}{c} \text{UHR}=\left(\frac{A_{i}/A_{f}}{A_{i}}\right). \end{array}$$

This index measures the ulcer re-epithelization [34].

### 2.14. Histological Evaluation

Skin tissue samples (*n* = 5) from treated and untreated lesions measuring 1 × 1 cm from all groups studied (PHP, PHP+, PHP−, silver sulfadiazine—positive control, and untreated—negative control) were collected and fixed immediately after collection in 10% buffered formalin. After fixation for a minimum period of 24 h, the samples were gradually dehydrated in hydroalcoholic solutions of increasing concentrations of ethanol (70%, 95%, and 100%) to remove water from the tissue. The dehydrated samples were then transferred to xylene to ensure an efficient transition to paraffin. Subsequently, the samples were infiltrated and embedded in melted paraffin, allowing the samples to be preserved and solidified, making them suitable for making ultrathin sections. Paraffin-embedded skin tissue samples were cut into ultrathin sections (5 μm thick) using a microtome. These sections were mounted on glass slides. Slides containing the ultrathin sections were selected for hematoxylin and eosin (HE) staining to highlight cellular structures and to analyze the new tissue formed as well as the advent of proliferation of inflammatory cells. The slides were subsequently mounted with coverslips and prepared for microscopic analysis. The slides were then analyzed using a light microscope (Axiolab, Carl Zeiss GmbH, Germany), and images were taken using an attached camera (DFC300FX, Leica Microsystems GmbH, Germany).

### 2.15. Collagen Content Assessment

Ultrathin section slides were also selected for Picrosirius red staining to highlight collagen fibers in the connective tissue of the animals [35] according to the experimental groups (PHP, PHP+, PHP-, silver sulfadiazine—positive control, and untreated, negative control group) (*n* = 5). The dye solution (0.1% in saturated picric acid) was applied to the tissue sections and then incubated for 1 h at room temperature, ensuring that the tissue sections were evenly covered with the staining solution. After staining, the slides were briefly rinsed with distilled water. The sections were washed with 0.5% acetic acid in 100% ethanol for 2–3 min to remove excess dye and differentiate the collagen fibers. The slides were rinsed again with distilled water to remove any remaining acetic acid. The tissue sections were dehydrated by passing them through a graded ethanol series (70%, 95%, and 100% ethanol) for 2–3 min each. After dehydration, clear the tissue sections in xylene or another suitable clearing agent (e.g., toluene) for 2–3 min. A mounting medium was then applied to the tissue sections and covered with a coverslip. Slides were then washed and mounted with coverslips for microscopic analysis. Slides stained with HE and Picrosirius red were examined under polarized light microscopy. Collagen fibers exhibit bright red/orange under polarized light, with type I collagen appearing in a more intense red/orange and type III collagen often appearing in a more greenish/yellow hue. The birefringence observed under polarized light helps distinguish between different collagen types based on their orientation and structure. During the analysis, the characteristics of the tissue were observed, including cellular morphology, the presence and quantification of cellular infiltrate, the presence of collagen fibers, vascularization, and any other relevant characteristics. This analysis of these characteristics, in terms of their morphological features, was qualitative, but to an extent that these characteristics could provide a differential analysis of the results.

Collagen quantification through Picrosirius red staining was performed using the open-source image processing software ImageJ calculating as a percentage of the area of each image (expressed in pixels). Initially, images of representative areas of the slides stained with Picrosirius red were captured using a Leica DM 2500 photomicroscope with the same lighting configuration and settings for all samples. Images were then loaded into ImageJ. Subsequently, the scale was defined so that the software could calculate measurements in real units. For this measurement, the “Analyze” function in the menu and selecting “Measure” were used to calculate the area of the selection that corresponded to the collagen in the image. This action was repeated in 10 fields of each slide in different areas. After collagen quantification in all images, the data were analyzed and comparison between the experimental and control groups was obtained.

### 2.16. Statistical Analysis

Graphics and statistical analyses were performed with GraphPad Prism Version 5 (La Jolla, CA, USA) and Statistic 10.0 (StatSoft®, Tulsa, OK, USA). For the fibroblast viability, data were presented in descriptive statistics presented as means ± SD and/or submitted to one-way analysis of variance (ANOVA), followed by the post hoc test of Tukey HSD (honest significant difference) for multiple comparisons, allowing comparison between different treatments and periods. The results were submitted to a log-rank test for the fibroblast proliferation curve to compare the proliferation curves according to the groups. One-way ANOVA and Dunnett's post hoc test were also used to analyze the results of the in vitro acute toxicity test—up and down. The results of the scratch test were analyzed using parametric Student's *t*-tests to evaluate the differences between the means between two groups (experimental x control group). ANOVA, Student's *t*-test, and nonparametric tests (Kruskal–Wallis) were used in the statistical analysis of the collagen content as the parametric data were not homogenous.

Statistical analysis used for WCP and UHR was Student's *t*-test, which is appropriate and most commonly used to assess differences between the means between two groups (experimental group x control group). The results of collagen content were statistically analyzed using one-way ANOVA followed by Tukey post-test. For histological analysis, comparison tests (Student's *t*-test) were used to identify statistically relevant variations in histological characteristics between groups. In addition, ANOVA and Tukey's test were applied when appropriate to assess the significance of differences between multiple groups. The established significance in all statistical analyses was 5%.

## 3. Results

### 3.1. FTIR Spectroscopy Analysis of the Chemical Bonds

**Figure 1:**
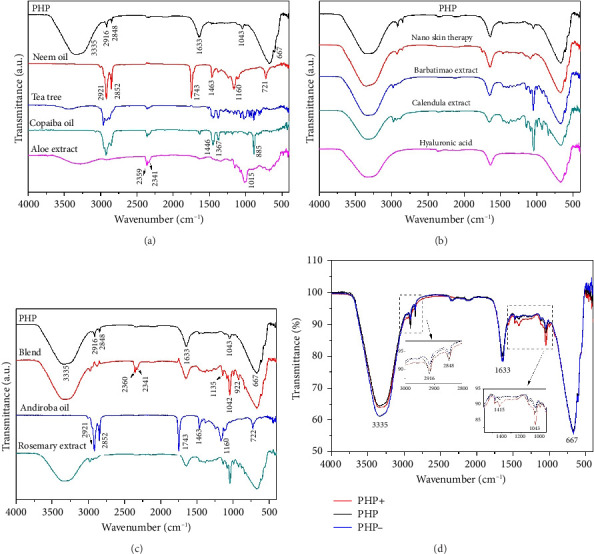
Representative FTIR spectra of samples: (a) PHP and bioactive ingredients ranging from 0.050 (aloe extract) to 0.400% (neem oil); (b) PHP and bioactive ingredients ranging from 0.500 (hyaluronic acid) to 1.000% (nano skin therapy); (c) PHP and bioactive ingredients ranging from 1.750 (rosemary extract) to 10.00% (blend); (d) ointment at different concentrations of bioactives (PHP+, PHP, and PHP−).

Infrared spectroscopy (FTIR) is a valuable analytical technique that offers a straightforward approach to identifying the presence of specific functional groups in materials. Each functional group exhibits characteristic vibrational frequencies that can be utilized to distinguish it from other groups [36]. FTIR was applied frequently for drug analysis during pharmaceutical preparation [37] and bio-pharmaceutical detection [38]. The bioactive spectra were grouped according to the range of their concentrations at the ointment standard formula (PHP). The exact concentration of bioactive ingredients was omitted to preserve the confidentiality of formulation. Thus, in Figure 1(a), a representative spectrum of PHP and the spectra of the bioactive ingredients present in lower concentrations in the product formulation (aloe extract, copaiba oil, tea tree essential oil, and neem oil) are presented.  Figure 1(b) illustrates the spectra of the PHP sample and the bioactive compounds present in intermediate concentrations (hyaluronic acid, Calendula extract, barbatimao extract, and Nano skin therapy). In Figure 1(c), the spectra of bioactives with higher concentrations in the product formulation (rosemary extract, andiroba oil, and blend) are also displayed and compared with the spectra of PHP, whereas Figure 1(d) illustrates the spectra of ointment samples at different concentrations of bioactives (PHP, PHP+, and PHP−). In addition, FTIR spectra show the characteristic vibration bands of samples.

Table 2 indicates the infrared frequencies and corresponding band assignments. All spectra related to extract samples as well as nano skin therapy, hyaluronic acid, blend, and ointment compositions showed a broad band referring to the -OH stretching between 3435 and 3280 cm^−1^, which indicates the presence of water. It is possible to check the characteristic vibration of -CH stretching in the region between 2979 and 2960 cm^−1^ for all extracts, except for aloe, and blend. The absorption bands in the 2920 and 2850 cm^−1^ regions are related to the asymmetric ν_asy_(CH_2_) and symmetric ν_sym_(CH_2_) vibrations, respectively, as well as the stretching of -CH_3_ groups. The strong absorbance band at 1743 cm^−1^ indicates that a carbonyl group (C=O) is present in neem oil and andiroba oil. Medium signals in the FTIR spectra observed in the region of 1647-1633 cm^−1^ are mainly related to the *ν*(C=C) stretching vibration. Still, in the range of 1463-1414 cm^−1^ and 1377-1338 cm^−1^, characteristic bands are observed representing the *δ*(C–H) bending vibration. Another characteristic of the spectra is the presence of peaks in the region between 1181 and 1015 cm^−1^, which can be attributed to the *ν*(C-O) stretching vibration. In the same way, the weak absorbance bands between 990 and 921 cm^−1^, the sharp peak at 885 cm^−1^, and the intense broadbands at ∼667 cm^−1^ are assigned to the *δ*(C=C) bending mode. Other bands (2360 and 2341 cm^−1^) seem to be related to technical artifacts (surface moisture adsorption by the specimens and the presence of CO_2_), which leads to results that might not representative of the sample being analyzed. All ointment formulations exhibited similar spectra, irrespective of the concentration of bioactives. No other peaks were observed, and this demonstrates that all samples have the same functional groups. However, the peak intensities at 2916 and 2848 cm^−1^, which are assigned to the *ν*(CH_2_) and *ν*(CH_3_) stretching vibrations, decrease as the concentration of bioactives decreases in the ointments. This is also observed considering the interval in the range of 1500-1000 cm^−1^. Furthermore, some of the bands observed in the spectra of the bioactive compounds were not identified in the ointment formulations. This can probably be attributed to the low concentration of some of the bioactive compounds.

### 3.2. Evaluation of % Cell Viability in Cultures Exposed to the Regenerative Ointment (XTT Analysis)

**Figure 2:**
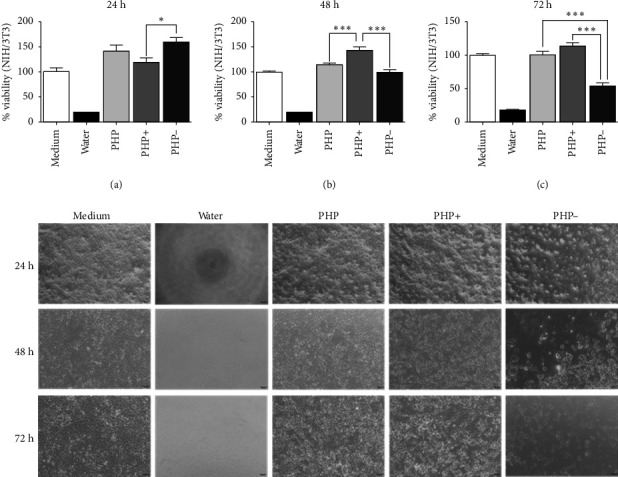
Percentage (%) of fibroblast viability (NIH/3T3) in indirect contact by the PHP, PHP+, and PHP− groups. The results are presented according to the mean ± standard deviation (SD) of the percentage values obtained in the XTT viability assay in the periods of (a) 24 h, (b) 48 h, and (c) 72 h. As a growth control, supplemented medium was used (medium group), and as a death control, distilled water was used (water group). Three independent experiments (*n* = 5) were performed. The statistical analysis of the data included a first evaluation of the normal distribution, followed by the one-way analysis of variance (ANOVA) following Tukey's test for multiple comparisons between groups. Below are representative images corresponding to each evaluated period (40x). It is possible to observe the cellular density in microscopic images. ⁣^∗^*p* = 0.01, and ⁣^∗∗∗^represents < 0.001.

**Figure 3:**
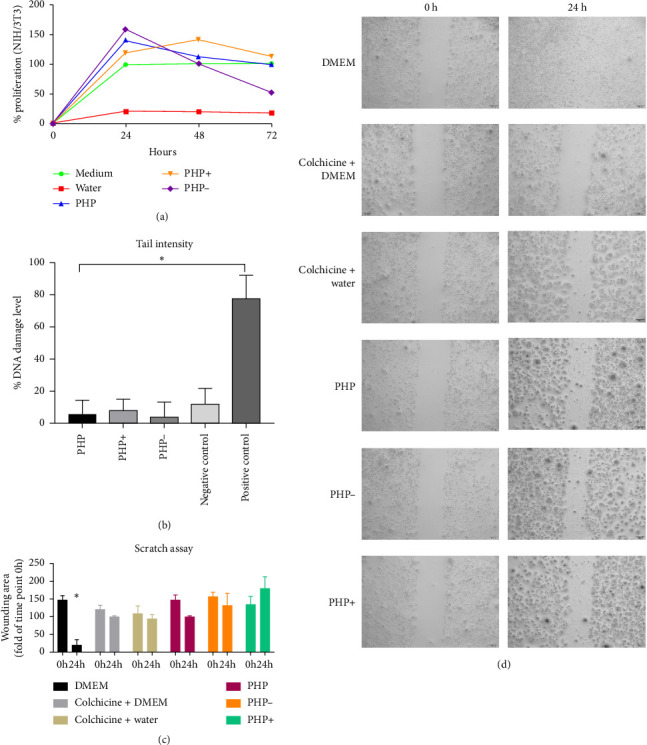
Evaluation of in vitro biocompatibility, bioactivity, and acute toxicity of the regenerative ointment. (a) Fibroblast proliferation curve (NIH/3T3) compared to the PHP, PHP+, and PHP groups. In the graph, the results of the percentages (%) and average values obtained in the viability test (XTT) in periods of 24, 48, and 72 h are plotted. As a negative control, supplemented DMEM medium was used (medium group), and as a positive control, distilled water was used (water group). Three independent experiments were performed. The parametric quantitative results were submitted to a log-rank test to compare the proliferation curves according to the groups. ⁣^∗^*p* = 0.01; ⁣^∗∗∗^*p* = 0.01. (b) Graph with the results of the genotoxicity study evaluated by the comet assay. The graph presents the level of DNA damage (tail intensity) caused to NIH/3T3 cells in indirect contact with all tested formulations. (c) The graph demonstrates that there were no significant differences among the studied groups; only the negative control showed a greater recovery area in the 24 h period. (d) Photomicrographs exhibit the wound healing in the cell monolayer lesion. The scale bar represents 200 μm.

In the cell viability analysis, the negative control group (medium group) exhibited similar mean percentage values (*p* > 0.05), close to 100%, across all evaluated time periods (24, 48, and 72 h). Likewise, the positive control group (water group) displayed the lowest mean values (approximately 19%) throughout the analysis, with no significant difference between them (*p* > 0.05). These findings validate the results obtained for the experimental groups, as depicted in Figures 2 and 3.

The study revealed that the regenerating ointment ensured a beneficial impact on cell viability, regardless of the experimental groups (PHP, PHP+, and PHP-), when in indirect contact with fibroblasts (NIH/3T3) for 24 h. The average cell viability percentage in the experimental groups (PHP, PHP+, and PHP-) was significantly higher (*p* < 0.05) compared to the negative control group (∼40%). Moreover, the viability exceeded the recommended threshold of ≥ 70% according to ISO 10993-5. Additionally, the experimental groups not only exhibited lower cytotoxicity but also demonstrated the ability to stimulate cell proliferation, with percentages ranging from 119.1% to 159.4% compared to the negative control group (99.8%) (Figure 2).

It is important to note that while the PHP group (with viability percentages of 140.9%, 113.1%, and 100.1% for the respective evaluation periods) and the PHP- group (with viability percentages of 159.4%, 101.3%, and 52.9% for the respective evaluation periods) showed a tendency of decreasing viability over time, reaching the lowest percentage at 72 h for the PHP- group (52.9%), the PHP + group exhibited a significant increase in cell viability (119.1% and 143% at 24 and 48 h, respectively) compared to the negative control and the 24-hour period of the same group (*p* < 0.001). Additionally, at 72 h, the cell viability still remained similar to that of the negative control group (*p* > 0.05), with a relative increase of 13.5% regardless of the statistical equivalence (Figure 2). Thus, PHP samples, mainly after 24 h of indirect contact (through medium conditioning) with fibroblasts, proved to be biocompatible and have relevant proliferative potential, according to this analysis. Analyses of cell morphology performed showed adherent cells with a flattened and elongated shape, which are characteristic of viable cells. This aspect was found in cells treated with PHP, PHP+, and PHP- conditioned medium, as can be seen in the representative images of Figure 2.

### 3.3. Genotoxicity Assay—Comet Test

The findings from the genotoxicity study revealed that none of the tested formulations (PHP, PHP+, and PHP−) induced significant DNA damage, which can potentially trigger apoptosis or result in mutations associated with the development of diseases linked to genetic alterations (Figure 3). The observed DNA damage is within the range of normal cellular processes and can be repaired naturally. Thus, all tested formulations exhibited satisfactory results in this analysis, as the level of DNA damage was comparable to or even lower than that of the untreated negative group.

### 3.4. Cell Migration Assay

The migration test did not demonstrate any improvement in the wound healing of the cell monolayer lesion. All groups under study exhibited similar results, except for the negative control group, which exhibited greater recovery of the cell monolayer within 24 h (Figure 3).

### 3.5. In Vitro Acute Toxicity Test—Up and Down

The cytotoxicity and genotoxicity results obtained from the evaluation of the cells in culture after 24 to 72 h of indirect contact with the conditioned medium of the three tested formulations revealed that none of the formulations exhibited cytotoxic or genotoxic effects. No lethal dose (LD) was found in the tested formulations, so PHP, PHP+, and PHP− demonstrated safety for use in cell cultures (Figures 2 and 3). As acute toxicity manifests within short periods (hours or days) after exposure, typically characterized by tissue breakdown or cell death, to degrees that exceed repair or adaptation of the biological system, often in these cases there is an observation of rapid lethal effects, which were not observed at any time in the in vitro tests performed. Conversely, in the up and down test, which allows the determination of cytotoxicity (OECD 129), the results were very positive, as even an overdose of the product was not able to cause harmful effects.

### 3.6. Wound Healing Evaluation in Rat Model of Skin Wound

**Figure 4:**
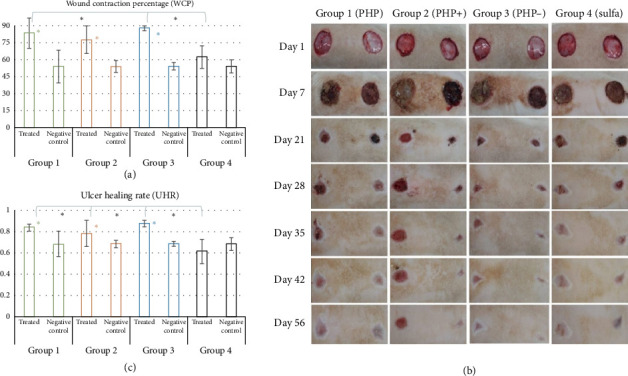
(a) Representation of the percentage (%) of wound contraction in all groups studied, demonstrating the contraction of the treated lesion and negative control (untreated), respectively. Statistical analysis revealed that PHP, PHP+, and PHP− presented significant means of WCP compared to that of their respective untreated control groups (*p* < 0.05). No significance was observed when the WCP of the group treated with the silver sulfadiazine (Group 4) was compared to that of its control untreated group. (b) Representative images of the development of the study, demonstrating the treated and untreated lesions of one animal from each group studied on days 1 (start of the study), 7, 21, 28, 35, 42, and 56. (c) Mean UHR of the groups studied, presenting treated and untreated lesions (negative control), respectively, for each group. Treatment with the formulations PHP, PHP+, and PHP− exhibited significantly higher means of UHR of the treated lesions when compared to their negative control (untreated) areas, with the reduction of the ulcerated area with consequent re-epithelialization (*p* < 0.05). All PHP formulations exhibited significant mean when compared to the positive control silver sulfadiazine. ⁣^∗^*p* < 0.05.

Representative images of the evolution of the closure of excisional skin wounds in animals are displayed in Figure 4. According to the observations of adverse reactions, none of the treated groups presented adverse responses in the first 30 days of treatment. Therefore, no skin irritation was observed after applying the treatments during this period. However, at the end of the experiment, close to the 42^nd^ day of treatment, slight skin irritation was observed around the treated area. The most affected animals belonged to experimental groups PHP, PHP+, and PHP−. Slight skin irritation typically resolves on its own within hours to a day after the irritating substance exposure and usually has no effect on wound healing [39]. Therefore, these adverse reactions ceased in the following week, progressing to normal skin at the end of the experiment. Regarding the local mild skin irritation around the treated area after application of formulations PHP, PHP+, and PHP−, as previously described, this reaction ceased progressing to normal skin at the end of the experiment. The observations allow us to outline some hypotheses to elucidate this effect. One of them is based on the sticky texture of the treatments used (ointments), which favors the accumulation of residues from the animal's maintenance box, especially in areas where hair has grown (areas adjacent to the lesion), due to greater adherence to the hair. This retention may have led to the “irritated” appearance that was observed at this point in the experiment.

### 3.7. Wound Contraction (WCP) and Ulcer Re-Epithelization (UHR)

The results of the percentage (%) of WCP and UHR in the macroscopic analysis obtained in the in vivo preclinical study are displayed in Figure 4. Groups PHP, PHP+, and PHP- presented an average wound contraction of 83.6%, 78.0%, and 88.0%, respectively. The WCP values observed in the positive control (sulfadiazine) and in the control untreated groups were, respectively, 62.0% and 57.0%. Statistical analysis revealed that PHP, PHP+, and PHP− presented significant means of WCP compared to that of their respective untreated control groups (*p* < 0.05). That means that the extent to which a wound contracted or closed over time was significantly higher when treated with PHP, PHP+, and PHP−. In other words, the groups treated with ointment formulations presented more fully contracted and/or closed wounds, with the edges coming together, and fewer visible gaps remained. In this manner, formulations PHP, PHP+, and PHP− caused no adverse responses to the animals and were effective in regulating the healing process, with higher wound contraction compared to the untreated control group. Ointment formulations PHP and PHP- exhibited significantly higher means compared to the positive control silver sulfadiazine (*p* < 0.05). When the mean of WCP of the group treated with the silver sulfadiazine was compared to that of its control, no significance was observed (*p* > 0.05).

Regarding the analysis of UHR, it was observed by comparing the ulcer area at day 1 and day 56 that groups PHP, PHP+, and PHP− healed at rates of 0.84, 0.78, and 0.88, respectively. Comparatively, the group treated with silver sulfadiazine had a healing rate of 0.62. Similarly, the rate at which the untreated ulcers healed at the same time point was 0.69 (Figure 4). Treatment with the formulations PHP, PHP+, and PHP− exhibited significantly higher means of UHR of the treated lesions when compared to their negative control (untreated) areas, with the reduction of the ulcerated area with consequent re-epithelialization (*p* < 0.05). All PHP formulations exhibited significant mean when compared to the positive control silver sulfadiazine (*p* < 0.05).

### 3.8. Histological Evaluation

**Figure 5:**
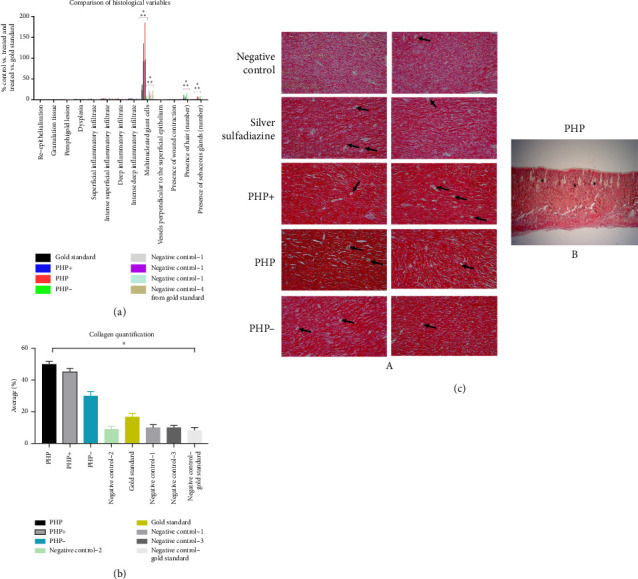
(a) Comparison of histological variables between the treated groups (PHP+, PHP, and PHP−) and the negative control and gold standard groups (silver sulfadiazine). Statistical significance is indicated by ⁣^∗^ symbols for comparisons between treated vs. treated groups. negative control group and ⁣^∗∗^ for comparisons between treated groups vs. the gold standard (*p* < 0.05). (b) The image displays the means of the quantitative analysis of collagen in the experimental groups using ImageJ. ⁣^∗^ Indicates statistical significance *p* < 0.05 treated group vs. negative control group and gold standard. (c) Histological plates from the study groups stained with Picrosirius red, where the variation in the intensity of the reddish coloring is visible, related to the amount of collagen (indicated in red in image A). Image B displays a histological slide of the PHP group, with the “⁣^∗^” representing blood vessels perpendicular to the tissue surface, one of the parameters evaluated. In spite of their increase observed in the PHP group, no significance was observed when the results of the experimental groups were compared. The arrows demonstrate the presence of giant cells in the tissue (image (c)). 20x magnification.

The histological analyses after 60 days revealed significant differences in the number of giant cells observed in the tissues (Figures 5(a) and 5(c)). In the groups treated with PHP and PHP+, it was observed a significant increase of, respectively, 190% and 120% in the number of giant cells compared to the positive (silver sulfadiazine) and negative control groups and PHP− (*p* < 0.05). Furthermore, a slight increase in the number of hairs and sebaceous glands was observed, with a tendency toward a greater increase being observed in the groups treated with the formulations PHP, PHP+, and PHP− compared to the negative and positive controls (Figure 5(a)).

Other variables studied, such as re-epithelialization, granulation tissue, pemphigoid lesion, dysplasia, superficial type inflammatory infiltrate (Sup type), deep type inflammatory infiltrate (Prof type), intense deep inflammatory infiltrate (Prof intens), vessels perpendicular to the surface epithelium, and presence of wound contraction exhibited no significant differences (*p* > 0.05) among the experimental groups (PHP, PHP+, PHP−, positive (silver sulfadiazine), and negative control groups) (Figure 5(a)).

### 3.9. Collagen Content Assessment

Regarding collagen quantification, the analysis revealed that lesions treated with PHP showed a significant difference compared to the control untreated group (*p* < 0.05), with a five-fold increase in the amount of collagen. When compared to the gold standard control group (silver sulfadiazine), a significant difference was also observed, whereas lesions treated with PHP treatment exhibited three times more collagen. Furthermore, collagen in this group was notably more organized compared to all groups analyzed (Figure 5(b)). Similarly, the group treated with PHP + also exhibited a significant increase in the amount of collagen (*p* < 0.05), with an increase of 4.5 times compared to the negative control groups and 2.7 times in relation to the gold standard—positive control group (Figure 5(c)). Although the PHP- group exhibited a smaller increase in the amount of collagen compared to other variations of the product Pharmacure + Classic Reestruturante, it was observed 3 times more collagen compared to the negative control group and 1.8 times higher compared to the gold standard control group (Figure 5).

## 4. Discussion

The investigation of natural compounds possessing anti-inflammatory, antioxidant, antibacterial, and collagen-synthesizing properties has shown promise in the field of wound healing [14]. The utilization of natural compounds offers several advantages over conventional approaches. Firstly, natural compounds are often readily available and can be obtained from various plant sources [40]. This accessibility contributes to their potential cost-effectiveness, making them viable options for widespread application in healthcare settings [41]. Additionally, natural compounds have demonstrated diverse biological activities, including anti-inflammatory effects that can help alleviate the inflammatory response associated with wound healing [18]. By reducing inflammation, natural compounds may promote a more favorable environment for the healing process to proceed [18]. Considering the sequential nature of the wound healing process and the intended use of the tested product, it is reasonable to expect that the natural bioactive compounds in combination with other compounds present in the formulation would exert their biological effects in alignment with the different phases of healing.

The healing process comprises three phases: hemostasis and inflammation; proliferation; and remodeling and maturation [17]. In the hemostasis phase, the first stage of wound healing, a sequence of biological events occurs, such as coagulation, platelet proliferation, expression of growth factors, cytokines, immunomodulation, collagen degradation, and edema formation. In this manner, based on the results, it seems that there was a synergistic biological effect provided by the ointment ingredients that supported and stimulated the wound healing due to the presence of bioactives from vegetable oils (andiroba and copaiba), of the essential oil of *Melaleuca alternifolia*, from plant extracts of *Carica papaya*, *Rosmarinus officinalis*, and *Stryphnodendron* sp., and also from Nano Skin Therapy and hyaluronic acid in the ointment composition[42–47]. Nano skin therapy is a commercial product containing bioactives from *Curcuma aromatica*, *Rosa aff rubiginosa*, and *Aloe vera* plant extracts nanoencapsulated in lipid particles with a diameter ≥ 200 nm. Nanoencapsulation allows the stabilization of sensitive and complex components that are highly soluble in water. These plant-based bioactives are also indicated for wound care [48–50].

Another ingredient in the formulation of the ointiment is a blend composed of glycolic extracts of *Carica papaya* and *Aloe vera *(Table 1). Aqueous propylene glycol extracts from medicinal plants are commonly used as active ingredients in the production of medicine and beauty products for external application [51]. Glycols are solvents used as extraction agents in the extraction of biologically active compounds from hydrophilic plant extracts [51]. It also acts as a carrier, enhancing the targeted delivery of natural ingredients. Glycols are used as humectants, reducing water activity, thus enhancing their preservative effect and allowing a long shelf life [52]. As bioactive ingredients, it is expected that these two glycolic extracts would present an enzymatic action in the wound prior to the granulation phase, helping in the formation of new scar tissue [15, 50].

In the proliferation phase, all the bioactives seem to act, whether in the proliferation of fibroblasts, collagen synthesis, angiogenesis, re-epithelialization, formation of granulation tissue, and wound contraction. Finally, in the remodeling phase and maturation of the scar tissue, it is expected that the copaiba oil, the plant extracts of *Aloe vera*, *Rosmarinus officinalis*, and *Stryphnodendron* sp., the essential oil of tea tree, and nano skin therapy act: inducing re-epithelialization, remodeling of collagen III to collagen I, stimulating the action of metalloproteinases, remodeling of fibroblasts to myofibroblasts, proliferation of keratinocytes, deposition of permanent extracellular matrix, and organization of the collagen fibers [42, 44, 50, 53, 54]. It is important to highlight that in the papaya glycolic extract contains glycolic papain, a proteolytic enzyme, which has enzymatic actions on the wound prior to the granulation phase, helping the formation of new scar tissue [55]. Another component to highlight is high-molecular-weight hyaluronic acid, which has the function of hydrating the wound and contributing to a humid microenvironment that also favors the formation of collagen [56].

Overall, the FTIR spectra analysis presented in Figure 1 provides information about the chemical composition, interactions, and potential synergistic effects of the product and its ingredients. The spectra presented in Figure 1 demonstrated the variations in the vibrational bands across different concentrations of bioactives and bioactive samples. The assignment of infrared frequencies and corresponding band assignments in Table 2 further aid in understanding the molecular bonds present in the samples. The spectrum of bioactive ingredients and ointment formulations exhibits several characteristic absorption peaks that are strongly affected by its physical and chemical structures [57]. For the FTIR spectrum of the PHP product, there is a broadband at 3335 cm^−1^. This band is assigned to the hydroxyl group (-OH) stretching vibrations. At 2916 and 2850 cm^−1^, two weak bands associated with the asymmetric and symmetric C–H stretching vibrations of CH_2_, respectively, and CH_3_ groups are observed. At 1640 cm^−1^, an absorption band related to the *ν*(C=C) stretching vibration is present. The weak band at 1043 cm^−1^ refers to the *ν*(C-O) stretching vibration. At 666 cm^−1^, an intense broadband is attributed to the *δ*(C=C) bending mode.

Interestingly, all PHP samples exhibit similar spectra, regardless of the concentration of bioactives, indicating the presence of the same functional groups. However, the peak intensities at 2916 and 2848 cm^−1^, corresponding to the *ν*(CH_2_) and *ν*(CH_3_) stretching vibrations, decrease as the concentration of bioactives decreases in PHP. This trend is also observed within the range of 1500-1000 cm^−1^. Furthermore, certain bands observed in the spectra of bioactive compounds are not identified in the PHP samples, likely due to the lower concentration of some bioactive compounds. These results provide valuable information about the molecular composition and structural variations in the PHP product and its bioactive components at different concentrations. Therefore, the FTIR method may yield richly structured ‘‘fingerprint” spectra relating to structure and conformation [58, 59]. Although the molecular composition of a wound dressing has a direct correlation with its biological activity by influencing key processes in wound healing, such as inflammation, infection control, tissue regeneration, and cell migration [60], further investigations are necessary to explore the implications of these variations and their potential impact on the efficacy and functionality of the ointment tested. Results of ongoing studies on proteomics will certainly provide a robust approach to profile and quantify proteins within cells, organs, or tissues, providing comprehensive insights about the dynamics of cellular processes, modifications, and interactions.

Virtually all plant-based bioactives in the formulation are able to synergistically act, either by stimulating, regulating, or inhibiting in different ways the complex and dynamic processes supported by countless cellular events, coordinated to effectively repair damaged tissue [61]. The exploration of natural compounds with anti-inflammatory, antioxidant, antibacterial, and collagen-synthesizing properties, in addition to the proteolytic enzyme effect of papain, represents a promising avenue for advancing wound healing strategies. The integration of these compounds into local solutions and ointments offers a cost-effective and accessible approach to wound care. However, further research is needed to elucidate the specific mechanisms of action, optimal formulations, and clinical efficacy of these natural compounds in wound healing. Continued investigation in this field holds the potential to revolutionize wound care practices and improve patient outcomes. The present study aimed to investigate the potential therapeutic effects of a regenerating ointment containing natural compounds in vitro, providing valuable insights for further research and development in wound care. The results demonstrated that the ointment positively influenced cell viability, exceeding the recommended threshold of 70% according to ISO 10993-5 guidelines (Figure 2). Notably, all tested formulations, including PHP, PHP+, and PHP-, exhibited no cytotoxicity or genotoxicity (Figures 2 and 3(b)). The genotoxicity study revealed that none of the formulations caused significant DNA damage or programmed cell death. The observed DNA damage levels, possibly broken chromosomes, micronuclei, or fragmented DNA, were within the range of normal cellular processes that can undergo repair. These findings suggest that the tested formulations are safe for use in living organisms, as their effects on cell cultures did not induce excessive cell death, membrane damage, or DNA damage. Moreover, the experimental groups stimulated the cell proliferative capacity (Figures 2 and 3(b)).

To further evaluate the safety profile of the regenerating ointment, an in vitro adaptation of the up and down test was conducted to simulate different doses administered in cell cultures (Figures 1(d) and 3). In the present study, an in vitro study was used as an alternative method for the median LD test, contributing to the reduction and refinement of the use of animals in research. In this sense, it was used as an adaptation of the test, simulating different doses administered in cell cultures (in vitro). The results showed that even the highest tested dose did not induce acute lethal effects in cell cultures. These results are encouraging, as they demonstrate the absence of harmful effects even at high doses, supporting the safety of the regenerating ointment.

Several medical approaches and therapeutic interventions can act on the different processes involved in the healing cascade by decreasing healing time, modifying inflammation, and accelerating the proliferative phase. In addition, some of these approaches can stimulate and optimize the remodeling phase, promoting more efficient recruitment of fibroblasts and collagen deposition [62, 63]. Therefore, considering that wound care represents a prominent clinical area among the different pathologies and is of great clinical importance, the correct treatment and management positively influence the course of healing, reducing the potential for complications. In this manner, the development of new products for wound care is of great importance and brings countless benefits to patients, reducing costs in public health and considerably improving the quality of life. The results of this in vitro study demonstrated that Pharmacure + Classic Reestruturante is safe, with no cytotoxic or genotoxic effects, and also enhances proliferative potential in cultured cells (Figures 3(a) and 3(b)), irrespective of its concentration.

Although the scratch migration tests did not reveal significant differences in wound closure in the cell monolayer between the ointment formulations (Figures 3(c) and 3(d)), the presence of PHP + composition appeared to induce wound opening, indicating its potential for effective debridement. It is worth noting that the lack of observed differences in the closure of the cell monolayer lesion reinforces the limitations of the in vitro scratch assay, which fails to replicate the intricate dynamics of a complete tissue composed of diverse cells that respond differently during the wound healing process.

Regarding the wound contraction, it was observed that all the ointment formulations (PHP, PHP+, and PHP−) presented higher means of wound contraction when compared to both the positive control group (sulfadiazine) and the control untreated groups (Figure 5). A high WCP, which means a significant decrease in wound area over time, generally indicates good healing and is considered a positive outcome in many clinical contexts [64]. Although a rapid or extensive wound contraction seems beneficial, it can have some clinical consequences depending on the situation, such as larger or deeper wounds [65]. Considering the results of Group 4 (positive control silver sulfadiazine), an increase in the wound contraction was also observed, but no significance was observed when compared to the control untreated group. In this manner, it can be speculated that the systemic release of the active ingredients of the sulfadiazine product rather than the local effects in this specific treatment might contribute to less wound contraction compared to the untreated groups (negative control).

The means of the UHR analysis also demonstrated that ulcers healed significantly faster with the treatment with the ointment formulations when compared at day 56. In this manner, ulcer re-epithelization was stimulated with treatment with PHP, PHP+, and PHP− when compared to both control groups. Ulcers were reported to significantly heal if the UHR is at least 0.40 [34, 66]. In this manner, all of the experimental groups achieved this milestone of re-epithelization, including the control groups. Systematic topical dressing is the most common form of treatment for ulcers, and 1% silver sulfadiazine has been more extensively used due to its potential antimicrobial activity [34]. In spite of this, the search for a product containing bioactives from natural compounds offers effective, sustainable, and safer alternatives for promoting wound healing, especially in low-income countries. Wound healing products with bioactives from medicinal plants are important for being cost-effective, sustainable, accessible, and culturally appropriate alternatives to expensive and hard-to-access medical treatments.

The results of the histological and morphological variables analyzed indicated that these variables were not affected by treatment with the different products and concentrations used. Therefore, regarding these specific characteristics, the treated groups demonstrated no significant improvement compared to the negative control groups. This lack of differences suggests that the treatment had no direct impact on re-epithelialization, the formation of granulation tissue, the presence of pemphigoid lesions, the occurrence of dysplasia, the types and intensity of inflammatory infiltrate, the formation of vessels perpendicular to the superficial epithelium, or wound contraction (Figure 5(a)). It is important to note that, although these variables presented no significant differences, they may play relevant roles in other aspects of the skin healing process, such as modulating inflammation, collagen formation, angiogenesis, scar formation, and other factors that contribute to the overall healing process and the quality of the final healed tissue. Therefore, it is essential to consider that the assessment of treatment efficacy must consider not only these isolated parameters but also other relevant clinical and biological results to obtain a complete picture of the therapeutic impact on the skin's healing and regeneration processes.

The significant increase in blood vessels perpendicular to the tissue surface indicates a response to the inflammatory or infectious process. However, it is important to note that no significant differences were identified between the groups studied in this aspect. This result suggests that although there was an increase in the formation of perpendicular vessels as part of the response to infection or inflammation (Figure 5(b)), this change was not exclusive to any specific treatment group, which may indicate that treatment with the different formulations of the product Pharmacure + Classic Reestruturante (PHP, PHP+, or PHP−) had no direct impact in this aspect. Therefore, other factors may be influencing this observed vascular response. It is important to further investigate these differences to understand the underlying mechanisms.

The correlation between collagen types and skin regeneration is fundamental in the context of developing new treatments to accelerate and stimulate skin regeneration [67]. Collagen plays an essential role in the structure and function of the skin, being the main component of the extracellular matrix [68]. There are several types of collagen, with type I collagen being the most abundant in the skin and playing a crucial role in maintaining tissue integrity and resistance [69, 70]. In the skin regeneration process, the formation and adequate organization of collagen are critical to ensuring the functional restoration of the tissue. Different types of collagen can play specific roles in skin healing and regeneration. For example, type III collagen is most commonly found in the initial phase of healing, while type I collagen is predominant in the remodeling phase [70]. Therefore, analyses of the types of collagen present in skin tissue, their organization, and their relative proportions are necessary to understand how these factors affect skin regeneration. This allows the development of specific treatments that aim to modulate collagen synthesis and organization to optimize the regeneration process. Understanding the relationships between collagen types and skin regeneration is crucial for the development of innovative therapies that promote more effective and esthetic skin recovery after injuries, traumas, or medical procedures.

The results observed in Figure 5(b) indicate that PHP, PHP+, and PHP− products play a positive role in stimulating collagen production, a key component in skin regeneration and healing. Organized collagen, especially evident in the PHP group, is a promising finding, as it suggests that this treatment not only increases the amount of collagen but also contributes to its proper organization, which is essential for the structural and functional integrity of tissues [67]. Therefore, this collagen analysis supports the efficacy of treatments with the product Pharmacure + Classic Reestruturante in promoting skin regeneration and improving the quality of scar tissue.

The increase in giant cells promoted by the treatment may be beneficial for accelerating the regeneration process due to their specialized function in the phagocytosis of foreign material and the removal of necrotic tissues. These cells are capable of cleaning the area of injury from cellular debris, bacteria, and waste, facilitating a cleaner environment conducive to tissue regeneration [71]. Furthermore, giant cells can secrete growth factors and cytokines that stimulate cell proliferation and angiogenesis, contributing to the rapid repair of damaged tissue. Therefore, the increase in these cells at the site of injury may play a crucial role in improving the skin's healing and regeneration processes [72, 73].

The results of the present in vivo preclinical study indicated not only the restructuring of the skin after injury but also the formation of complex appendages, such as hair and sebaceous glands, which play a crucial role in recovering the function of the regenerated tissue [74]. This increase in the presence of giant cells and the formation of attachments suggests a more effective response in the process of removing cell debris and necrotic tissues, which contributes to a healthier and pinker appearance of the wound bed in the groups treated with the product in comparison with the negative and positive control groups. Therefore, histological analyses corroborate the macroscopic findings and reinforce the effectiveness of the treatment in promoting healing and regeneration of skin tissue (Figures 5(a), 5(b), and 5(c)). These results indicate that PHP, PHP+, and PHP− formulations appear to play a positive role in stimulating collagen production, a fundamental component in skin regeneration and healing. Organized collagen, especially evident in the PHP group, is a promising finding, as it suggests that this treatment not only increases the amount of collagen but also contributes to its adequate organization, which is essential for the structural and functional integrity of tissues [67]. The increase in collagen production during wound healing is a key factor that directly correlates with faster healing and stronger tissue formation [69]. Collagen is the main structural protein in the extracellular matrix, and it plays an essential role in tissue repair and the restoration of skin and other tissues after injury [75]. The comprehension of the correlation between collagen synthesis and healing outcomes helps explain how improved collagen production contributes to better and faster wound healing [69]. This collagen analysis supports the effectiveness of treatments with the Pharmacure + Classic Reestruturante product (PHP, PHP+, and PHP−) in promoting skin regeneration and improving the quality of scar tissue.

It is important to emphasize that the main focus of the present study was to assess the safety and effectiveness of a wound healing ointment (named Pharmacure + Classic Reestruturante) containing bioactive ingredients derived from medicinal plants (extracts, essential oils, and vegetable oils). This product, PHP, contains the standard concentration of the commercial product. Two other concentrations were tested, PHP+ (three times higher bioactives than the standard) and PHP− (three times lower bioactives than the standard), for comparative reasons. According to the manufacturer, the fact that these two variations were different from that of the commercial product might lead to changes in the balanced formulation somehow affecting the physical, chemical, or biological properties, making it harder to ensure that these formulations are consistent with each other and the standard version. While this in vitro study demonstrated the safety and enhanced proliferative potential of the regenerating ointment, further investigations are warranted to confirm its effectiveness and safety in complementary studies to deeply understand how the orchestrated and complex nature of the biological actions provided by the regenerating ointment performs in the healing process. To address these limitations, additional research is also necessary to assess their long-term effects, potential interactions with other wound healing processes, and overall clinical impact. In this manner, additional testing in more complex human-like models, chronic wound conditions, and long-term follow-up studies would be necessary for a more accurate and comprehensive evaluation of wound healing products. A proteomic analysis of proteins expressed after treatment with an ointment will be performed in order to search for detailed insights into the biological effects of the product, also helping to understand its mechanism of action, potential benefits, and safety risks. In this manner, exclusive proteins expressed in the treated group (PHP) may represent potential targets for future therapies that aim to modulate the inflammatory process, increase collagen production, and perform other biological actions, thus improving healing results. In spite of this limitation, the in vitro and in vivo preclinical evaluations of the regenerating ointment tested in the present study demonstrated its safety and potential to enhance cell viability and proliferation. These findings provide a foundation for further research and development of the regenerating ointment, with the ultimate goal of improving wound care practices and patient outcomes.

## 5. Conclusions

The results of the present in vitro and in vivo studies revealed the superior healing effects of the regenerative ointment containing bioactive ingredients compared to those of the control group (silver sulfadiazine) and the control untreated group, particularly according to the results observed after the application of the product in standard-sized ulcers in the in vivo study. These findings collectively suggest that the tested ointment formulations possess a high degree of biocompatibility and pose no immediate cytotoxic, genotoxic, or acute toxic risks. Morphological analysis demonstrated that the ointment formulations caused no adverse responses, irrespective of the bioactive concentrations, and were effective in the healing process, reducing the ulcerated area with re-epithelialization. Although ulcers may heal on their own, our findings strengthen the view that the real value of the use of the regenerating ointment containing bioactives lies in the treatment of chronic ulcers that are generally unresponsive to conventional therapies. The exploration of natural compounds with specific biological properties represents a promising approach for advancing wound healing strategies.

## Figures and Tables

**Table 1:** Plant-based bioactives in the product formulation⁣^∗^.

1. Vegetable oils
Andiroba oil (*Carapa guianensis*)
Neem oil (*Azadirachta indica*)
Copaiba oil (*Copaifera langsdorffii*)
2. Plant extracts
Aloe dried extract (*Aloe vera*)
Rosemary glycolic extract (*Rosmarinus officinalis*)
Calendula glycolic extract (*Calendula officinalis*)
Barbatimao glycolic extract (*Stryphnodendron* sp.)
Blend (glycolic extracts of *Carica papaya* and *Aloe vera*)⁣^∗∗^
3. Essential oil
Tea tree (*Melaleuca alternifolia*)
4. Other bioactives
Nano skin therapy⁣^∗∗∗^
Hyaluronic acid
Blend (glycolic extracts of *Carica papaya* and *Aloe vera*)⁣^∗∗∗^

⁣^∗^Manufacturer's information.

⁣^∗∗^Pharmacure, Belo Horizonte, Brazil.

⁣^∗∗∗^Nanovetores Sapiens Parque, Florianópolis, Brazil.

**Table 2:** Infrared frequencies and band assignments of PHP samples and bioactive compounds.

Wavenumber (cm^−1^)														Band assignment
Bioactive compounds											PHP compositions			
Neem oil	Tea tree	Copaiba oil	Aloe extract	Nano skin therapy	Barbatimao extract	Calendula extract	Hyaluronic acid	Blend	Andiroba oil	Rosemary extract	PHP−	PHP	PHP+	
—	3435	—	3280	3350	3324	3324	3324	3312	—	3324	3335	3335	3335	*ν*(O-H)

—	2960	—	—	—	2978	2974	—	2979	—	2979	—	—	—	*ν*(C-H)

2921	2914	2924	2916	2924	—	2934	—	—	2921	—	2916	2916	2914	ν_asy_(CH_2_) *ν*(CH_3_)

2852	2875	2856	—	2853	—	2880	—	2882	2852	—	2850	2850	2848	ν_sym_(CH_2_) *ν*(CH_3_)

—	2360	2359	2359	—	—	—	2359	2360	—	2362	—	—	—	*ν*(O=C=O)

—	2341	2341	2341	—	—	—	—	2341	—	—	—	—	—	*ν*(O=C=O)

1743	—	—	—	—	—	—	—	—	1743	—	—	—	—	*ν*(C=O)

—	—	1633	1633	1643	1644	1647	1633	1642	—	1644	1633	1640	1640	*ν*(C=C)

1463	1446	1446	—	1462	—	—	—	—	1463	—	—	—	1414	*δ*(C-H)

1377	1377	1367	1338	—	—	—	—	—	1377	—	—	—	—	*δ*(C-H)

1160	—	1181	1148	—	—	—	—	—	1160	—	—	—	—	*ν*(C-O)

—	—	—	—	—	1136	1136	—	1135	1116	1136	—	—	—	*ν*(C-O)

—	—	—	—	1085	1081	1079	—	1079	—	1080	—	—	—	*ν*(C-O)

—	1026	—	1015	—	1042	1040	—	1042	—	1041	1043	1043	1043	*ν*(C-O)

—	—	967	—	—	990	990	—	990	—	—	—	—	—	*δ*(C=C)

—	—	—	—	—	921	921	—	922	—	921	—	—	—	*δ*(C=C)

—	887	885	895	—	—	837	—	—	—	—	—	—	—	*δ*(C=C)

721	—	—	—	—	—	—	—	—	722	—	—	—	—	*δ*(C=C)

—	—	—	667	667	669	667	667	667	—	669	667	666	667	*δ*(C=C)

*Note:ν* = stretching vibration, ν_asy_ = asymmetric stretching, ν_sym_ = symmetric stretching, and *δ* = bending vibration.

## Data Availability

The data that support the findings of this study are available on request from the corresponding author. The data are not publicly available due to privacy or ethical restrictions.
